# Ouabain Effects on Human Anaplastic Thyroid Carcinoma 8505C Cells

**DOI:** 10.3390/cancers14246168

**Published:** 2022-12-14

**Authors:** Mariana Pires Teixeira, Natalia Ferreira Haddad, Eliza Freitas Passos, Marcelle Novaes Andrade, Maria Luisa Arantes Campos, Joyle Moreira Carvalho da Silva, Camila Saggioro de Figueiredo, Elizabeth Giestal-de-Araujo, Denise Pires de Carvalho, Leandro Miranda-Alves, Luciana Souza de Paiva

**Affiliations:** 1 Laboratório de Imunorregulação, Departamento de Imunobiologia, Instituto de Biologia, Universidade Federal Fluminense, Niterói 24210-201, Brazil; 2 Programa de Pós-Graduação em Patologia, Universidade Federal Fluminense, Niterói 24220-900, Brazil; 3 Laboratório de Endocrinologia Experimental-LEEx, Instituto de Ciências Biomédicas, Universidade Federal do Rio de Janeiro, Rio de Janeiro 21941-902, Brazil; 4 Programa de Pós-Graduação em Endocrinologia, Universidade Federal do Rio de Janeiro, Rio de Janeiro 21941-170, Brazil; 5 Laboratório de Fisiologia Endócrina, Instituto de Biofísica Carlos Chagas Filho, Universidade Federal do Rio de Janeiro, Rio de Janeiro 21941-902, Brazil; 6 Programa de Pós-Graduação em Farmacologia e Química Medicinal, Universidade Federal do Rio de Janeiro, Rio de Janeiro 21941-912, Brazil; 7 Departamento de Neurobiologia e Programa de Pós-Graduação em Neurociências, Instituto de Biologia, Universidade Federal Fluminense, Niterói 24210-200, Brazil; 8 National Institute of Science and Technology on Neuroimmunomodulation—INCT-NIM, Fundação Oswaldo Cruz, Rio de Janeiro 21040-360, Brazil

**Keywords:** cardiotonic steroids, cardiac glycosides, ouabain, anaplastic thyroid cancer, cytokines

## Abstract

**Simple Summary:**

Anaplastic thyroid carcinoma is a rare and very aggressive thyroid carcinoma. Current conventional treatments for anaplastic thyroid carcinoma are not very effective, so there is an urgent need for new types of treatment for this disease. Some molecules known as cardiotonic steroids (e.g., ouabain), which were previously used for the treatment of patients with heart failure, appear to have anti-tumoral effects and cancer therapy potential. The aim of the present work was to analyze the effects of ouabain in human anaplastic thyroid carcinoma, using an anaplastic thyroid cell line (called 8505C) as the model. With this work, we hope to contribute to the study of the potential anti-tumoral roles of ouabain in the search for alternative anaplastic thyroid carcinoma treatments.

**Abstract:**

Anaplastic thyroid carcinoma (ATC) is a rare, but aggressive, carcinoma derived from follicular cells. While conventional treatments may improve patients’ survival, the lethality remains high. Therefore, there is an urgent need for more effective ATC treatments. Cardiotonic steroids, such as ouabain, have been shown to have therapeutic potential in cancer treatment. Thus, we aimed to evaluate ouabain’s effects in human anaplastic thyroid cells. For this, 8505C cells were cultured in the presence or absence of ouabain. Viability, cell death, cell cycle, colony formation and migratory ability were evaluated in ouabain-treated and control 8505C cells. The expression of differentiation and epithelial-to-mesenchymal transition (EMT) markers, as well as IL-6, TGFb1 and their respective receptors were also quantified in these same cells. Our results showed that ouabain in vitro decreased the number of viable 8505C cells, possibly due to an inhibition of proliferation. A reduction in migration was also observed in ouabain-treated 8505C cells. In contrast, decreased mRNA levels of PAX8 and TTF1 differentiation markers and increased levels of the N-cadherin EMT marker, as well as IL-6 and TGFb1, were found in ouabain-treated 8505C cells. In short, ouabain may have anti-proliferative and anti-migratory effect on 8505C cells, but maintains an aggressive and undifferentiated profile.

## 1. Introduction

Anaplastic thyroid cancer (ATC) is an extremely aggressive cancer derived from thyroid follicular cells. In ATC, thyroid follicular cells have an undifferentiated phenotype, are not dependent on thyroid stimulating hormone (TSH) stimulation and do not have the capacity to take up iodine [1]. Moreover, they usually do not express thyroglobulin (TG) or thyroid transcription factor 1 (TTF1). In fact, in ATC samples, thyroglobulin immunohistochemistry was negative in 96% of cases and for TTF1 immunohistochemistry it was negative in 70% of cases, while paired box gene 8 (PAX8) immunohistochemistry was positive in more than 54% of cases [2].

ATC is relatively rare, representing only 1–2% of all thyroid cancers; however, it accounts for 14–39% of thyroid cancer deaths, with a median overall survival of approximately 4–6 months [1,3]. The conventional treatment for ATC includes surgery, radiotherapy and chemotherapy. The choice for a patient’s treatment depends on multiple factors, such as the extent of the disease, the patient’s airway status, the tumor´s resectability and the patient´s goals of care. The surgical approach most used in ATC patients is total thyroidectomy with lymph node dissection. Radiotherapy is often given after surgery; nevertheless, in some cases, radiotherapy could be given before surgery to the patient. Chemotherapy may also be indicated and involves the use of agents that target cell division, DNA repair or the DNA structure. In non-surgical cases, radiotherapy and chemotherapy could be indicated [4,5,6]. It is important to highlight that, even though these approaches have been shown to improve a patient´s outcome, they normally fail in the long run. 

More recently, the identification of molecular alterations of ATC opened the possibility for the use of targeted therapies. In fact, the use of dabrafenib in combination with trametinib, which are BRAF and MEK inhibitors, is already approved by the U.S. Food and Drug Administration agency (FDA) for ATC patients with a BRAF V600E mutation [2,7]. Moreover, a clinical report from 2017 that indicated an improvement of a 65-year-old Caucasian woman with ATC suggested the use of multi-target therapy with sunitinib, a multi-target tyrosine kinase inhibitor; foscarnet, a broad-spectrum antiviral that reduces the bioactivity of fibroblast growth factor 2; and low-molecular-weight heparin, an anticoagulant that was shown to limit metastatic progression in patients with dysregulation of fibroblast growth factors/the fibroblast growth factor receptor pathway [8]. Even though new treatments for ATC have been under study, over the last few years little progress has been made. The development of new effective drugs for cancer treatment involves a lot of research, money and time. For those reasons, drug repurposing (or repositioning), which is a new therapeutic application for a previously clinically approved drug, could be an alternative strategy [9,10,11]. In this sense, cardiotonic steroids, such as ouabain, have shown the potential to be repurposed. 

Ouabain is a cardenolide, classically known as an inhibitor of Na+/K+-ATPase. Owing to its inotropic positive action and the resultant increase in cardiac muscle contraction, ouabain was used for many years as a treatment for congestive heart failure [12,13,14]. Several ouabain anti-cancer effects in human cell lines, such as non-small-cell lung cancer cell line A549, cervical cancer cell line Hela, colorectal carcinoma cell line HCT116 [15], renal cancer cell line OS-RC-2, small-cell lung cancer cell line NCI-H446 [16], breast cancer cell line MCF-7 [17] and glioma cell line U373MG [18], were already described. Nevertheless, not much is known about ouabain’s effects on human thyroid carcinomas. A recent work from our group has shown an in vitro anti-tumoral role of ouabain in the human papillary thyroid carcinoma cell lines [19]. Furthermore, a quantitative high-throughput drug screening previously published by Zhang et al. indicated an anti-proliferative activity of ouabain in different thyroid carcinoma cell lines [20]. Furthermore, a work by Tesselaar et al. has shown that digitalis-like compounds were able to restore sodium iodide symporter (NIS) expression and iodide uptake in the 8505C and Cal-62 human anaplastic thyroid carcinoma cell lines [21].

Therefore, the aim of the present work was to study the effects of ouabain on the 8505C human anaplastic thyroid carcinoma cell line’s viability, cell cycle, colony formation, cell differentiation, aggression in vitro and growth in vivo. Moreover, since it is well known that ouabain can modulate the production and secretion of cytokines in numerous in vivo and in vitro models, we also aimed to evaluate a possible modulation of interleukin 6 (IL-6) and transforming growth factor beta 1 (TGFβ1), cytokines that are involved in carcinogenesis, by ouabain.

## 2. Materials and Methods

### 2.1. Cell Culture

The 8505C human anaplastic thyroid cell line was used throughout this work. In some experiments, NTHY-ori, a non-tumor lineage, was also used for comparison. The 8505C and NTHY-ori cells were cultured in low glucose Dulbecco’s Modified Eagle’s Medium (DMEM) (Sigma-Aldrich, Saint Louis, MO, USA) supplemented with 10% fetal calf serum (FCS) (Gibco by Thermo Fisher Scientific, Waltham, Massachusetts) in an atmosphere of 5% CO_2_ at 37 °C. Both cell lines were kindly provided by Dr. Corinne Dupuy (Institut Gustave Roussy, Villejuif, France).

### 2.2. Cell Viability Assay

Cells were seeded in 96-well plates (Sarstedt, Numbrecht, North Rhine-Westphalia, Germany) at a concentration of 5 × 10^3^ cells/well in a final volume of 200 µL. Following an incubation period of 24 h at 37 °C in an atmosphere of 5% CO_2_, culture supernatants were removed and cells were cultivated in culture medium in the absence or presence of different concentrations (10^−5^–10^−11^ M) of ouabain (Sigma-Aldrich). Cell viability was evaluated with an MTT [3-(4,5-dimethylthiazol-2-yl)-2,5-diphenyltetrazolium bromide] assay as previously described [19], after 24 h of treatment with ouabain.

### 2.3. Cell Count

As described in our previous work [19], 5 × 10^5^ cells/well were seeded in 12-well plates (Sarstedt) in a final volume of 1 mL and incubated for 24 h in an atmosphere of 5% CO_2_ at 37 °C. Culture supernatants were then removed and fresh culture medium alone (control) or culture medium plus 10^−7^ M of ouabain was added to the respective wells. After 24 h of treatment, cells were trypsinized, centrifuged at 300× *g* for 5 min and pellets were resuspended in phosphate-buffered saline (PBS). Finally, cells were diluted in a trypan blue solution (Sigma-Aldrich) and counted with a Neubauer chamber.

### 2.4. Cell Death Analysis

As described in our previous work [19], 2 × 10^4^ cells/well were seeded in 24-well plates (Sarstedt) in a final volume of 1 mL. After the initial 24 h of incubation in an atmosphere of 5% CO_2_ at 37 °C, culture supernatants were removed and fresh culture medium alone (control) or culture medium plus 10^−7^ M of ouabain was added, after which cells were furthered incubated for 24 h. Cell death analysis was then performed according to the manufacturer’s instructions using a Muse Annexin V & Dead Cell Kit (Millipore, Burlington, MA, USA).

### 2.5. Cell Cycle Analysis

As described in our previous work [19], 5 × 10^5^ cells were seeded in 35 mm culture dishes (Sarstedt) in a final volume of 2 mL. Following 24 h of incubation in an atmosphere of 5% CO_2_ at 37 °C, culture supernatants were removed and replaced with fresh culture medium (control) or culture medium plus 10^−7^ M of ouabain, and cells were cultivated for an additional 24 h. Cell cycle analysis was then performed according to the manufacturer’s instructions using a Muse Cell Cycle Kit (Millipore).

### 2.6. Reactive Oxygen Species (ROS)

A total of 5 × 10^4^ cells/well (final volume of 1 mL) was seeded in 12-well plates (Sarstedt). Following the initial 24 h of incubation in an atmosphere of 5% CO_2_ at 37 °C, culture supernatants were removed and cells were incubated for another 24 h in fresh culture medium without phenol red in the presence or absence of 10^−7^ M of ouabain. Intracellular ROS levels were evaluated using chloromethyl 2′,7′-dichlorodihydrofluorescein diacetate (CM-H2DCFDA) (Invitrogen by Thermo Fisher Scientific). Briefly, cells were trypsinized and loaded with 2 µM of CM-H2DCFDA in Hank’s Balanced Salt Solution (Sigma-Aldrich) in the dark for approximately 30 min at 37 °C. H_2_O_2_ (200 µM, Sigma-Aldrich) was used as a positive control. The excess dye was removed with centrifugation and cells were suspended in PBS. Fluorescence intensity was assessed in a BD FACSCalibur™ flow cytometer (BD Biosciences, Franklin Lakes, NJ, USA).

### 2.7. Tumor Cell Colony-Forming Units (CFUs)

A total of 5 × 10^4^ cells/well was seeded in 12-well plates (Sarstedt) and incubated for 24 h in an atmosphere of 5% CO_2_ at 37 °C. Culture supernatants were removed and cells were incubated for an additional 24 h with fresh culture medium alone or culture medium plus 10^−7^ M of ouabain. Untreated or ouabain-treated cells were then trypsinized and centrifuged at 300× *g* for 5 min, the pellets were resuspended in culture medium, and the cells were counted with a Neubauer chamber. Next, 102 NTHY-ori cells or 103 8505C cells were seeded in 6- and 12-well plates (Sarstedt), respectively. Plates were incubated for a further 7 days for the analysis of the colonies (clusters containing 50 cells or more) that arose after cell treatment. Each experiment was performed independently at least four times in triplicate.

### 2.8. Transwell Cell Migration Assay

A total of 10^5^ cells/well was seeded in 6-well plates (Sarstedt) and incubated for 24 h in an atmosphere of 5% CO_2_ at 37 °C. Supernatants were then removed and cells were incubated for an additional 24 h with fresh culture medium alone or culture medium plus 10^−7^ M of ouabain. Untreated or ouabain-treated cells were trypsinized and centrifuged at 300× *g* for 5 min, the pellets were resuspended in culture medium, and cells were counted with a Neubauer chamber. A total of 5 × 10^4^ 8505C cells in 500 µL of DMEM containing 1% FCS were added to the upper chambers of transwell inserts (8 µm pore membrane filters) and 750 µL of DMEM containing 5% FCS was added to the lower chambers. Plates were then incubated for 24 h for cell migration. Control and ouabain-treated 8505C cell migration were analyzed as previously described by Teixeira et al. [19]. A transwell migration assay was performed independently four times in duplicate.

### 2.9. Real-Time PCR

As described in our previous work [19], 10^5^ cells/well were seeded in 6-well plates (Sarstedt) in a final volume of 2 mL and incubated for 24 h in an atmosphere of 5% CO_2_ at 37 °C. Next, culture supernatants were removed and cells were cultured in the presence or absence of 10^−7^ M of ouabain for 24 h. Total RNA was isolated using TRI Reagent^®^ solution (Sigma-Aldrich). To ensure the removal of genomic DNA from RNA preparations, we used DNASE I and RNase-free (Thermo Fisher Scientific, Waltham, Massachusetts, EUA), and cDNA was synthesized using a High-Capacity cDNA Reverse Transcription Kit (Applied Biosystems, Waltham, MA, USA), according to the manufacturer’s protocol. mRNA levels of the target genes were evaluated with real-time polymerase chain reaction (qPCR) on a 7500 real-time PCR system (Applied Biosystems) using GoTaq^®^ qPCR Master Mix (Promega, Madison, WI, USA). Each sample was amplified in triplicate and relative expression levels of target genes were calculated using the 2^−ΔΔct^ method [22]. β-Actin was used as the internal control. Primer pairs (5′-3′) for interleukin-6 (IL-6), interleukin-6 receptor (IL-6R), transforming growth factor beta 1 (TGFb1), transforming growth factor beta receptor type I (TGFbRI), transforming growth factor beta receptor type II (TGFbRII), vimentin, twist-related protein 1 (TWIST1), matrix metallopeptidase 9 (MMP9), N-cadherin, paired box gene 8 (PAX8), thyroid transcription factor 1 (TTF1) and β-actin are described in Supplementary Table S1. 

### 2.10. Western Blot

A total of 5 × 10^5^ cells/well was seeded in 6-well plates (Sarstedt) in a final volume of 2 mL and incubated for 24 h in an atmosphere of 5% CO_2_ at 37 °C. Culture supernatants were removed and cells were cultured in the presence or absence of 10^−7^ M of ouabain for 24 h. After this, cultures were homogenized in a RIPA lysis buffer. Lysates were centrifuged at 10,000× *g* for 10 min at 4 °C, and the protein concentration was determined using a BCA assay. Western blot procedures were performed as described previously [23]. Samples with 30 μg protein per lane were resolved in 8–10% SDS-PAGE gels and blotted onto nitrocellulose membranes using a semi-dry blotting system (Bio-Rad Laboratories, Hercules, CA, USA). Membranes were blocked with 5% (*w*/*v*) BSA or 5% non-fat dry milk (*w*/*v*) in TBS-T (20 mM Tris-HCl, 160 nM NaCl, 0.1% Tween-20) for 2 h at room temperature. Primary antibodies, rabbit polyclonal anti-phospho-mTOR (1:1000), rabbit polyclonal anti-mTOR (1:1000), rabbit polyclonal anti-phospho-AMPKα (Thr 172) (1:1500), rabbit polyclonal anti-AMPKα1 (1:1000), rabbit polyclonal anti-phospho-AMPKβ (1:1000) and rabbit polyclonal anti-AMPKβ1/2 (1:1000) (Cell Signaling Technology, Danvers, MA, USA) were diluted in blocking solution and incubated with the membranes overnight at 4 °C. After washing 3 times (10 min) with TBS-T, membranes were incubated with HRP-conjugated secondary antibody goat anti-rabbit (1:20,000) diluted in TBS-T for 1 h at room temperature and imaged in a ChemiDocL-Pix System (Loccus Biotecnologia^®^, Cotia, São Paulo, Brazil) using Luminata reagent. After stripping with 0.1 M glycine-HCl buffer (pH 2.2) for 10 min at room temperature, membranes were blocked as described above and re-probed with a rabbit monoclonal anti-GAPDH (1:1000), after which they were incubated with the same HRP-conjugated secondary antibody mentioned above. Images were analyzed using ImageJ software (NIH, Bethesda, MD, USA). GAPDH was used as a loading control protein.

### 2.11. In Vivo Treatment with Ouabain

Balb/c nude mice were obtained from Universidade Federal Fluminense (Brazil) and housed in a germ-free temperature-controlled room with water and food provided ad libitum. Nude mice were sub-cutaneously injected with approximately 107 8505C cells. On the 21st day post-injection, mice were divided into two groups: the control group, in which mice were daily inoculated intraperitoneally (i.p.) with 200 µL/mouse of vehicle (PBS); and the OUA group, in which mice were daily inoculated i.p. with 200 µL/mouse of ouabain 10^−7^ M. Mice were weighed and tumor volumes were measured once every three days. Tumor volumes were calculated using the formula volume = (width^2^ × length)/2. After 15 days of ouabain treatment, mice were euthanized and tumors were removed for weighing.

### 2.12. Statistical Analyses

Statistical analyses of data were performed using GraphPad Prism 5 (GraphPad Software, Inc.). A D’Agostino-Pearson omnibus test and/or Shapiro–Wilk test was used as normality tests in order to evaluate data distribution. Statistical tests used in this study are mentioned in figure legends and values of *p* < 0.05 were considered statistically significant.

## 3. Results

### 3.1. Ouabain Decreased the Number of Viable Cells in 8505C Cell Cultures

**Figure 1:**
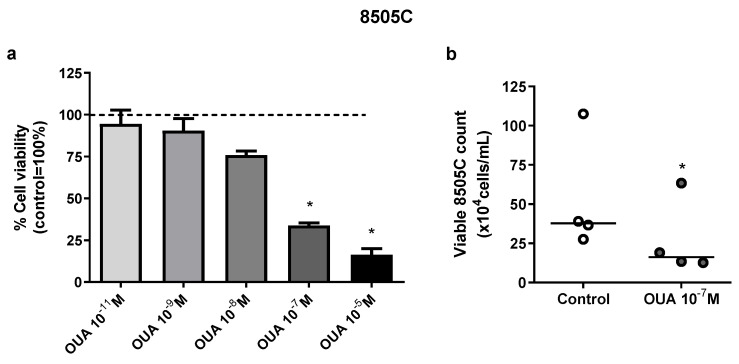
Ouabain decreased 8505C viability in vitro. 8505C cells were cultured in the absence or presence of ouabain at concentrations that ranged from 10^−5^–10^−11^ M for 24 h. Next, cell viability was evaluated with MTT assay or using cell counting in a Neubauer chamber using trypan blue. (**a**) MTT absorbance values of ouabain-treated cultures were normalized to their respective control and data are presented as the mean relative percentage of viable 8505C cells ± SEM after 24 h of culture. (**b**) Scatter plots represent the absolute number of viable 8505C cells with reference lines showing the medians. At least three independent experiments were performed for each condition. * *p* < 0.05 using repeated measures ANOVA with Dunnett´s post-test and paired *t*-test, respectively.

The viability of 8505C in the presence or absence of ouabain (10^−11^–10^−5^ M) was evaluated after 24 h of culture. A significant decrease in 8505C viability was observed in MTT assays after incubation with 10^−5^ and 10^−7^ M of ouabain for 24 h (Figure 1a). Similarly, in trypan blue exclusion tests, a decrease of approximately 50% in the number of viable 8505C cells was also found in cultures treated with 10^−7^ M of ouabain when compared to control cultures (Figure 1b). 

### 3.2. Ouabain Did Not Increase 8505C Apoptosis, Necrosis or Reactive Oxygen Species (ROS) Levels

**Figure 2:**
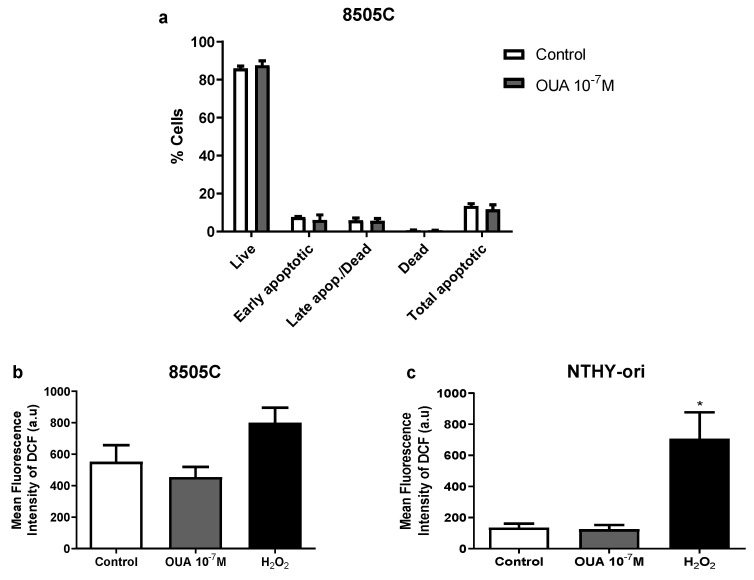
Ouabain did not increase 8505 death or ROS levels. 8505C and NTHY-ori cells were cultured for 24 h with 10^−7^ M of ouabain or left untreated. (**a**) 8505C death was analyzed using Muse Annexin V & Dead Cell Kit (n = 3). Data are expressed as the mean percentage of live, early apoptotic, late apoptotic/dead and total apoptotic cells ± SEM in ouabain-treated and control cultures. (**b**,**c**) 8505C and NTHY-ori intracellular ROS levels (CM-H2DCFDA fluorescence) were evaluated with flow cytometry (n = 6). Data are expressed as the mean fluorescence intensity of DCF in 8505C (**b**) and NTHY-ori (**c**) cells. * *p* < 0.05 using repeated measures ANOVA with Dunnett´s post-test.

Next, cell death analyses were performed in 8505C cultures in an attempt to explain the decreased viability previously observed in cultures treated with 10^−7^ M of ouabain. No significant differences were found between 8505C ouabain-treated and control cultures after 24 h regarding the percentage of live, apoptotic and necrotic cells when using a Muse Annexin V & Dead Cell Kit (Figure 2a).

Then, we analyzed reactive oxygen species (ROS) levels, because ROS production is an important mechanism of cytotoxicity to cancer cells. No significant difference in mean fluorescence intensity of dichlorofluorescein (DCF) was observed when comparing ouabain-treated and control cells after 24 h of culture, suggesting that ROS levels were not affected by ouabain treatment (Figure 2b,c). 

### 3.3. Ouabain Induced G2/M Cell Cycle Arrest and Decreased Colony-Forming Units in 8505C and NTHY-Ori Cell Cultures

**Figure 3:**
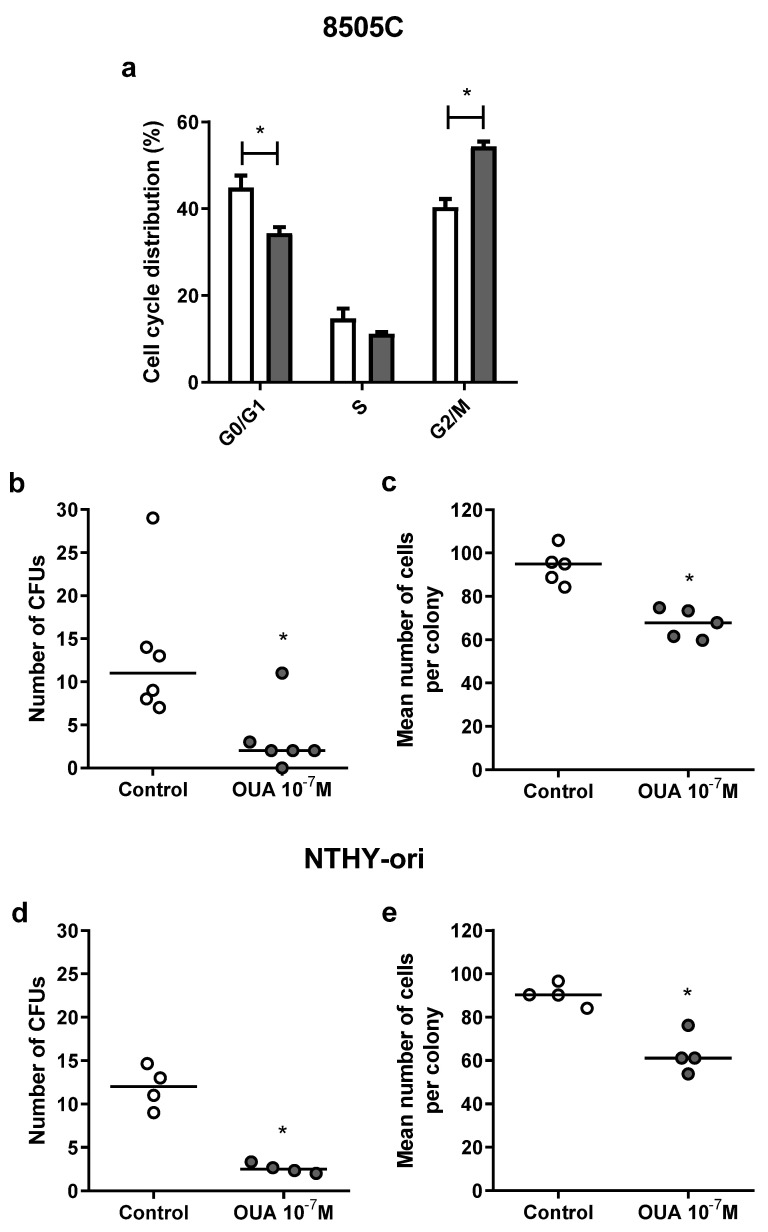
Effect of ouabain on 8505C cell cycle distribution and 8505C and NTHY-ori CFU formation. 8505C and NTHY-ori cells were cultured in the absence or presence of ouabain 10^−7^ M. (**a**) Cell cycle distribution was analyzed using Muse Cell Cycle Kit. Data are expressed as the mean percentage of 8505C cells ± SEM in G0/G1, S and G2/M phases after 24 h of culture (n = 4). (**b**–**e**) Tumor cell colony-forming units were also analyzed after 7 days of culture. Graphs are presented as the number of CFUs and the mean number of cells per colony in 8505C ((**b**,**c**), respectively) and NTHY-ori ((**d**,**e**), respectively) ouabain-treated and control cultures (n ≥ 4). * *p* < 0.05 using paired *t*-test.

In another attempt to explain the decreased viability in 8505C cell cultures treated with 10^−7^ M of ouabain, cell cycle analyses were performed using a Muse Cell Cycle Kit. In these analyses, a significant decrease in the percentage of 8505C cells in the G0/G1 phase along with an increase in the G2/M phase (Figure 3a) was observed after 24 h of incubation with ouabain. 

Moreover, to study the impact of ouabain in cell proliferation, we assessed cells’ clonogenic ability and evaluated the number of ouabain-treated and control cells’ colony-forming units (CFUs), as well as the mean number of cells of CFUs. As shown in Figure 3b–e, ouabain decreased the number of CFUs and the mean number of cells of these CFUs in 8505C (Figure 3b,c, respectively) and NTHY-ori (Figure 3d,e, respectively) cultures after 24 h. 

### 3.4. Ouabain Decreased 8505C Migration

**Figure 4:**
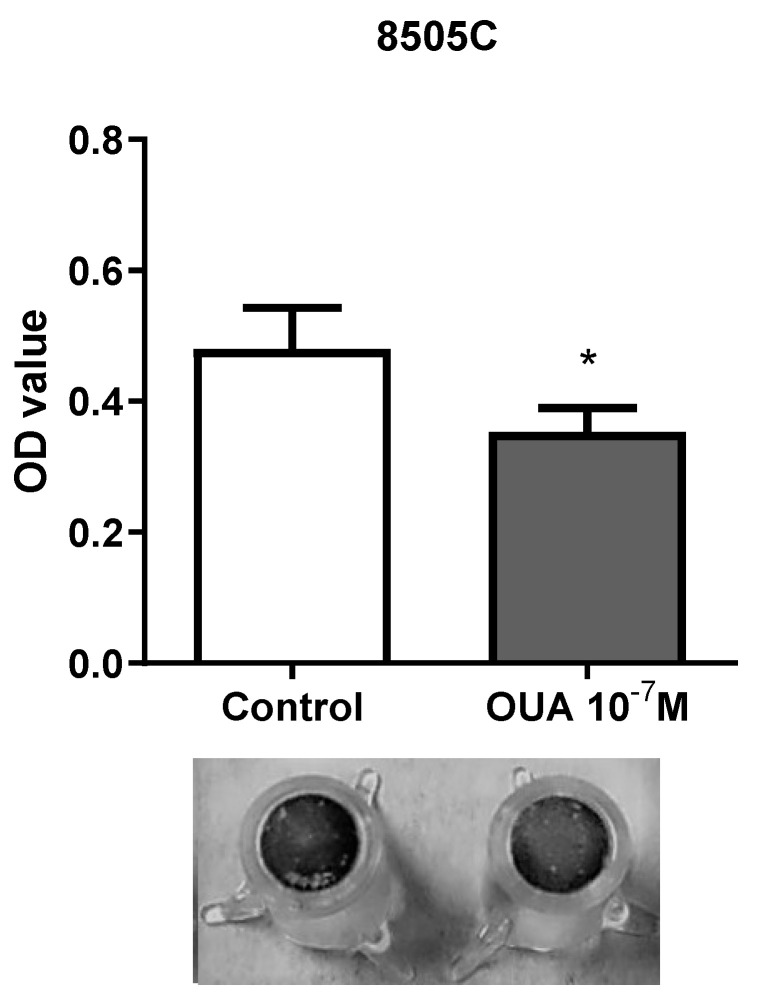
Ouabain decreased 8505C migration ability. 8505C cells were cultured in the absence or presence of 10^−7^ M of ouabain for 24 h and then allowed to migrate through transwell inserts for 24 h, as described in Materials and Methods section. Bar graph displays transwell migration measurement as mean OD ± SEM of four independent experiments performed in duplicate. A representative image of transwell inserts with 8505C control and ouabain-treated migrating cells stained with crystal violet is also shown. * *p* < 0.05 using paired *t*-test.

Since ouabain appeared to inhibit the proliferation of 8505C, we then decided to evaluate cell migration using transwell assays. Our results demonstrated that ouabain reduced the migration of 8505C (Figure 4). 

### 3.5. Ouabain Decreased PAX8 and TTF1 mRNA Expression in 8505C Cells

**Figure 5:**
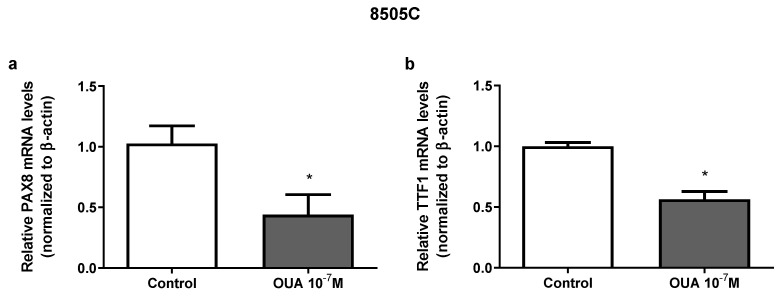
Effect of ouabain on mRNA expression of PAX8 and TTF1 in 8505C cells. Following 24 h of 8505C culture in the absence or presence of ouabain 10^−7^ M, PAX8 and TTF1 mRNA levels were evaluated by RT-PCR. (**a**,**b**) Data are presented as the mean ± SEM of mRNA levels of PAX8 (**a**) and TTF1 (**b**) of at least three independent experiments, each performed in triplicate. * *p* < 0.05 using unpaired *t*-test.

To investigate ouabain’s effects on cell differentiation, paired box gene 8 (PAX8) and thyroid transcription factor 1 (TTF1) mRNA expression was evaluated. Thyroid stimulating hormone receptor (TSHR) mRNA expression was also evaluated, but was not detected in our experiments due to a low expression of this gene. As shown in Figure 5, the treatment with 10^−7^ M of ouabain for 24 h induced a significant reduction in both the PAX8 (Figure 5a) and TTF1 mRNA levels (Figure 5b). 

### 3.6. Ouabain Modulated the Expression of Epithelial to Mesenchymal Transition Markers in 8505C Cells

**Figure 6:**
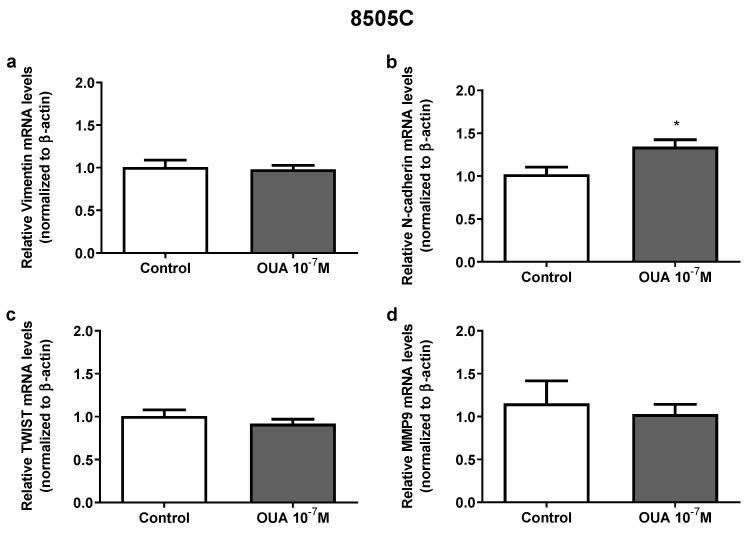
Effect of ouabain on aggressiveness markers expression in 8505C cells. Following 24 h of 8505C culture in the absence or presence of ouabain 10^−7^ M, aggressiveness markers expression was evaluated by RT-PCR. (**a**–**d**) Data are presented as the mean ± SEM of mRNA levels of Vimentin (**a**), N-cadherin (**b**), TWIST (**c**) and MMP9 (**d**) of at least three independent experiments, each performed in triplicate. * *p* < 0.05 using unpaired *t*-test.

Previously, results from our group showed that 10^−7^ M of ouabain modulates the expression of vimentin, TWIST and MMP9 in papillary carcinoma TPC-1 and BCPAP human thyroid cell lines [16]; however, in 8505C cells, only an increase in N-cadherin mRNA expression was observed in ouabain-treated cells after 24 h (Figure 6a–d).

### 3.7. Ouabain Increased IL-6 and TGFβ1 mRNA Expression in 8505C Cells

**Figure 7:**
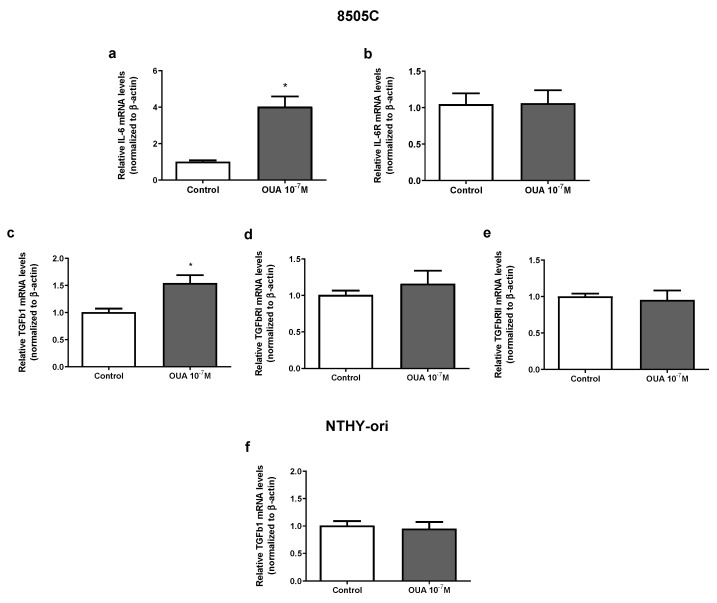
Effect of ouabain on mRNA expression of IL-6, IL-6R, TGFβ1 and TGFRs in 8505C and NTHY-ori cells. Following 24 h of 8505C culture in the absence or presence of ouabain 10^−7^ M, IL-6, TGFβ1 and their respective receptors expression were evaluated using RT-PCR. (**a**–**e**) Data are presented as the mean ± SEM of mRNA levels of IL-6 (**a**), IL-6R (**b**), TGFβ1 (**c**), TGFRI (**d**) and TGFRII (**e**) in control and ouabain-treated 8505C cells. (**f**) Data are presented as the mean ± SEM of mRNA levels of TGFβ1 in control and ouabain-treated NTHY-ori cells. For this, at least four independent experiments were performed in triplicate. * *p* < 0.05 using unpaired *t*-test.

IL-6 and TGFβ appeared to impact thyroid cancer tumorigenesis. Previous results from our group demonstrated that ouabain (10^−7^ M) increased IL-6 and IL-6R mRNA levels in TPC-1, BCPAP (papillary carcinoma human thyroid cell lines) and NTHY-ori (non-tumoral human thyroid cell line) after 24 h of culture [19]. An increase in IL-6 mRNA levels was also found in ouabain-treated 8505C cells, but IL-6R mRNA levels were not affected (Figure 7a,b, respectively). Similarly, TGFβ1 mRNA levels, but not TGFβ receptors type I and II, were augmented in ouabain-treated 8505C cells (Figure 7c–e, respectively). No significant difference was observed in TGFβ1 mRNA levels in ouabain-treated and control NTHY-ori cells (Figure 7f).

### 3.8. Ouabain Did Not Significantly Affect mTOR and AMPK Signaling Pathways

**Figure 8:**
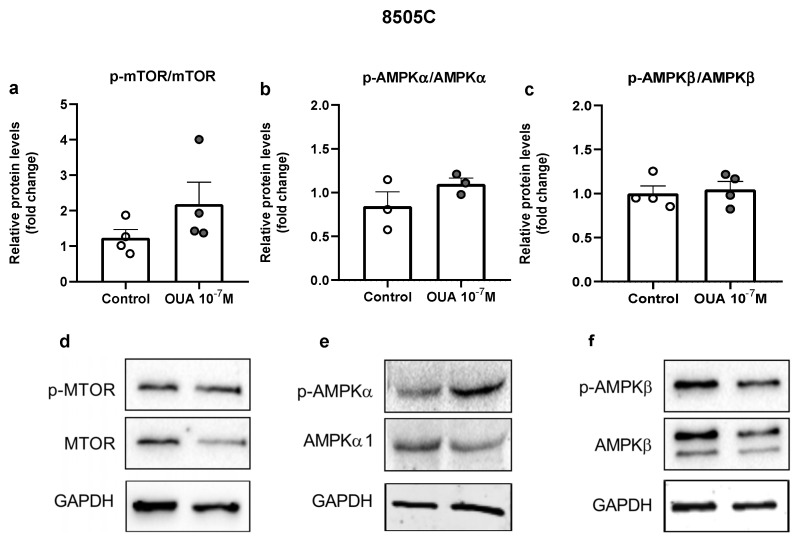
Impact of ouabain on mTOR and AMPK expression in 8505C cells. 8505C cells were cultured in the absence or presence of 10^−7^ M of ouabain and, after 24 h, protein levels of mTOR, p-mTOR, AMPKα1, p-AMPKα, AMPKβ and p-AMPKβ were evaluated. (**a**–**c**) Graphs show relative p-mTOR/mTOR (**a**), p-AMPKα/AMPKα1 (**b**) and p-AMPKβ/ AMPKβ (**c**) protein levels in control and ouabain-treated 8505C cells. (**d**–**f**) Representative blots of at least three independent experiments.

In order to evaluate the mechanism of action of ouabain in 8505C cells, we evaluated the mammalian target of rapamycin (mTOR) and adenosine mono-phosphate-activated protein kinase (AMPK) signaling pathways. No significant differences were observed in p-mTOR/mTOR, p-AMPKα/AMPKα or p-AMPKβ/AMPKβ ratios when ouabain-treated 8505C cells were compared to control cells; nevertheless, a tendency of increase was found in the p-mTOR/mTOR ratio (Figure 8). 

### 3.9. Ouabain In Vivo Treatment

**Figure 9:**
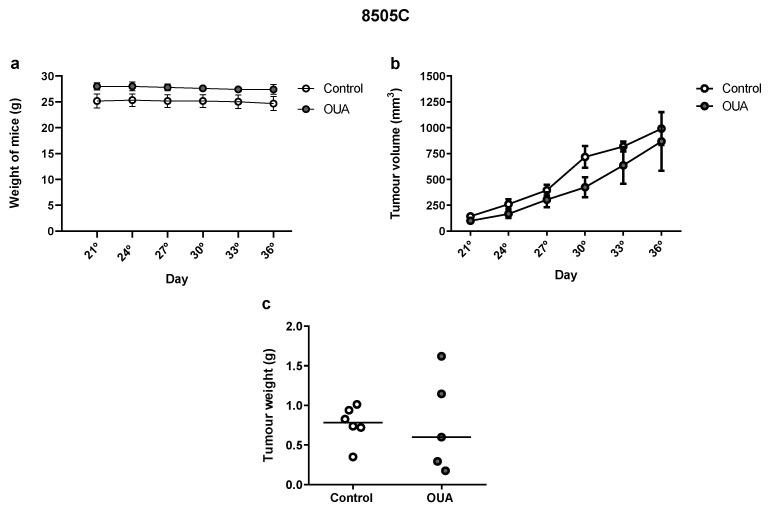
In vivo effect of ouabain. Balb/c nude mice were sub-cutaneously injected with ≈ 107 8505C cells. From the 21st day post-injection, mice were inoculated intra-peritoneally daily with vehicle or ouabain (10^−7^ M) for 15 days. (**a**,**b**) Graphs show mean weights (**a**) and tumor volumes (**b**) ± SEM measured once every three days. (**c**) Scatter plot represent the tumor weights after 15 days of treatment with reference lines showing the medians.

Finally, we evaluated the effect of ouabain in the in vivo growth of 8505C cells in Balb/c nude mice. Our results showed no significant differences in mice weight (Figure 9a), tumor volume (Figure 9b) or tumor weight (Figure 9c) when ouabain-treated mice were compared to the controls.

## 4. Discussion

In this study, we observed a decreased number of viable cells in ouabain-treated 8505C cell cultures after 24 h. This decrease could not be explained by an increase in cell death, as shown by the absence of changes in the percentages of live, apoptotic or necrotic cells and ROS levels in ouabain-treated 8505C cells. In fact, it appears that ouabain decreased cell growth, as indicated by the induction of cell cycle arrest at the G2/M phase and the decrease in colony formation in ouabain-treated 8505C cells. Similar results were found in ouabain-treated NTHY-ori cells, as demonstrated in our previous [19] and present work. These data were also corroborated with a previous work by Zhang et al., which showed that ouabain inhibited 8505C growth and induced cell cycle arrest [20]. 

In our previous work [19], we demonstrated that ouabain decreased cell migration and the expression of epithelial to mesenchymal transition (EMT) markers associated with tumor cell aggressiveness, such as vimentin, SNAIL-1 and MMP-9, in the human thyroid papillary cancer TPC-1 and BCPAP cell lines. In the present work, we also observed a decrease in the migration of ouabain-treated 8505C. However, ouabain did not affect the expression of the same EMT markers and even promoted an increase in N-cadherin mRNA expression in 8505C cells. Additionally, decreased mRNA levels of the thyroid differentiation cell markers PAX8 and TTF1 were found in ouabain-treated 8505C cells. These data suggest that ouabain treatment could lead to a more aggressive and undifferentiated phenotype of this human thyroid anaplastic cancer cell line, but with decreased migratory capacity. It is well-known that TTF-1 and PAX-8 are responsible for thyroid organogenesis and thyrocyte differentiation; nevertheless, some studies have already demonstrated a role of these molecules in cell proliferation and migration. Christophe-Hobertus et al. previously observed that the inhibition of TTF-1 activity due to the expression of a functional antagonist of TTF-1 decreased 8505C cell proliferation and the expression of mRNAs encoding positive effectors (such as CDK1 and cyclinB1), and increased the expression of mRNAs encoding negative regulators of cell division (such as CDKN2B and DUSP6) [24]. On the other hand, Dupain et al. described that TTF-1 can affect not only proliferation, but also migration and tumorigenicity in thyroid cancer cells. In the ARO anaplastic thyroid carcinoma cell line, the upregulation of TTF-1 increased the cell doubling time, cell migration and in vivo cell growth (in a nude mice model) [25]. Our results showed a decrease in TTF1 mRNA levels and an increase in N-cadherin mRNA levels in ouabain-treated 8505C cells. Moreover, decreased migration was observed in these cells. The upregulation of N-cadherin, concomitant with the downregulation of E-cadherin, is one of the hallmarks of epithelial-to-mesenchymal transition and is usually associated with increased proliferation, migration and invasion [26,27]. However, it is worth mentioning that there is a consensus that EMT status cannot be based on one molecular marker [28]. Taking everything into account, it is possible that ouabain’s effect in the proliferation and migration of 8505C cells could be a consequence of the decreased expression of TTF1, regardless of the increased expression of N-cadherin.

In order to better understand ouabain’s effects in 8505C cells, we evaluated the expression of IL-6 and TGFβ1 cytokines and their receptors in control and ouabain-treated cells. It has previously been shown that IL-6, in the human anaplastic thyroid cancer HTh74 and HTh74R (doxorubicin-resistant) cell lines, did not significantly affect cell viability (in MTT assay), but augmented colony formation. In addition, IL-6 diminished E-cadherin and increased vimentin and snail expression [29]. On the other hand, TGFβ1 was shown to inhibit the growth of the human anaplastic thyroid cancer HTh74, HTh 83, C643 and KAT-4 cell lines [30]. However, in 8505C cells, TGFβ1 inhibition with small RNA interference reduced cell proliferation, migration and invasion, but increased cell apoptosis. Decreased in vivo growth was also observed with TGFβ1 inhibition [31]. TGFβ1 also appears to impact thyroid differentiation; TGFβ1 has been shown to decrease PAX8 gene transcription, but not TTF1, in the FRTL-5 rat thyroid cell [32]. Similarly, TGFβ1 decreased PAX8 mRNA and protein levels in the PCCL3 rat thyroid cell [33]. Moreover, TGFβ1 was shown to increase the expression of EMT markers N-cadherin and vimentin in the non-small-cell lung cancer cell line [34]. In our work, we observed that ouabain increased IL-6 and TGFβ1 mRNA expression in 8505C cells, but did not affect their respective receptors. In NTHY-ori cells, ouabain increased both IL-6 and IL-6R mRNA expression [19], but did not impact TGFβ1. In TPC-1 and BCPAP, ouabain also increased both IL-6 and IL-6R mRNA expression [19]. Taking this into account, it is possible that some of the effects promoted by ouabain in 8505C cells and the differences in response observed between these thyroid cell lines could be related to these cytokines.

In an attempt to unravel the cell-signaling mechanisms of ouabain in 8505C cells, mTOR and AMPK pathways were studied. It is well known that AMPK has a key role in intracellular energy homeostasis and it is believed that AMPK can inhibit tumor cell growth through the suppression of the mTOR pathway [35]. It has previously been shown that TGFβ1 and IL-6 increased mTOR phosphorylation in human mesangial cells [36]. TGFβ1 can also trigger phosphorylation of mTOR in the human trophoblast HTR-8/SVneo cell line [37]. TGFβ1 induced AMPK phosphorylation in the human lung cancer A549 and H1460 cell lines [38]. In 8505C cells, heat shock protein 90, in combination with histone acetyltransferase inhibitor triptolide, reduced cell viability and increased cell death, possibly through the inactivation of mTOR pathway [39]. Similarly, the combination of baicalein and docetaxel, and glioma-associated oncogene antagonist 61, also appeared to induce anti-cancer effects in 8505C cells through the suppression of the mTOR pathway [40,41]. Contrastingly, metformin diminished the viability of 8505C and induced a slight increase in apoptosis; however, in this case, total mTOR expression was increased [42]. Ouabain has been shown to modulate many cell signaling pathways, including mTOR and AMPK. In this regard, ouabain (0.05, 2.5, 25 μM) was able to suppress U-87MG human malignant glioma cell line growth and motility after 24 h treatment through an inhibition of the Akt/mTOR signaling pathway. However, it is important to highlight that mTOR and p-mTOR expression levels were not affected by 0.5 μM of ouabain [43]. Moreover, 25 nM of ouabain decreased the expression of phosphorylated 4EBP1, an mTOR substrate, in the H460 human NSCLC cell line after 24 h of treatment. On the other hand, an increase in p-AMPK in ouabain-treated H460 cells was observed in this study [44]. Ouabain has also been shown to activate the AMPK signaling pathway in the MCF7 human breast cancer cell line under the same conditions (treatment with 25 nM of ouabain for 24 h) [45]. In the present study, only a tendency for a p-mTOR/mTOR ratio increase was observed in 8505C cells treated with ouabain (10^−7^ M or 100 nM). This difference could be explained by the use of different concentrations of ouabain (25 vs. 100 nM) or by differences in response to ouabain for distinct cell lines.

## 5. Conclusions

In conclusion, our results show that in vitro treatment with ouabain, at a concentration of 10^−7^ M, decreases the viability, colony formation and migration of the human anaplastic thyroid 8505C cell line. On the other hand, an in vitro treatment with ouabain also decreased the levels of PAX8 and TTF1, and increased the N-cadherin, IL-6 and TGFβ1 mRNA levels, while in vivo treatment did not significantly impact 8505C growth in Balb/c nude mice. Nevertheless, the exact mechanisms involved in these ouabain effects in 8505C cells remain to be elucidated. 

## Data Availability

The data presented in this study are available on request from the corresponding author.

## Supplementary Materials

The following supporting information can be downloaded at https://www.mdpi.com/article/10.3390/cancers14246168/s1: Table S1: Primers used for real-time PCR assay, product size and GenBank accession number.
