# Supplementary material for: Ouabain Effects on Human Anaplastic Thyroid Carcinoma 8505C Cells
Source: Cancers (Basel). 2022 Dec 14;14(24):6168. doi: 10.3390/cancers14246168 (PMC9777381; doi:10.3390/cancers14246168)
Supplement: Supplementary file 1 [file cancers-14-06168-s001.zip › cancers-2101677 WB original images.pdf]

a

Western blot whole membranes

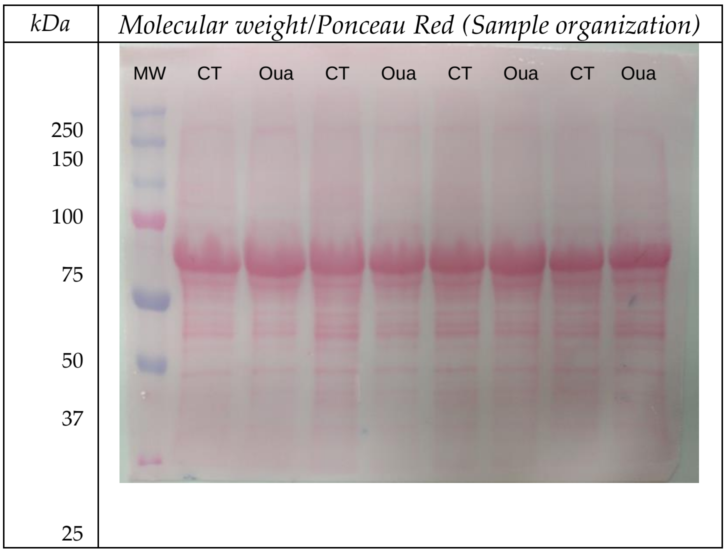

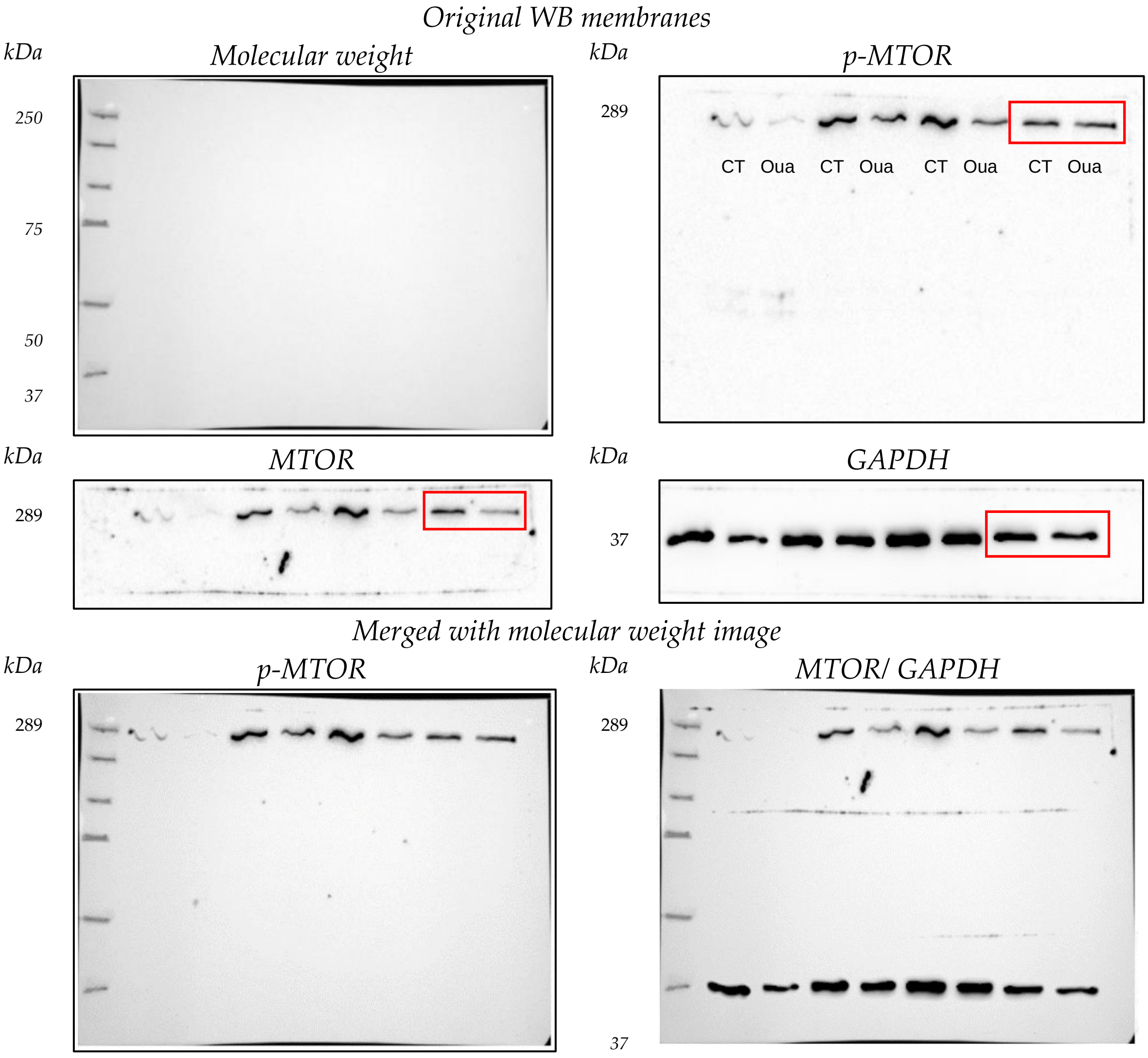

b

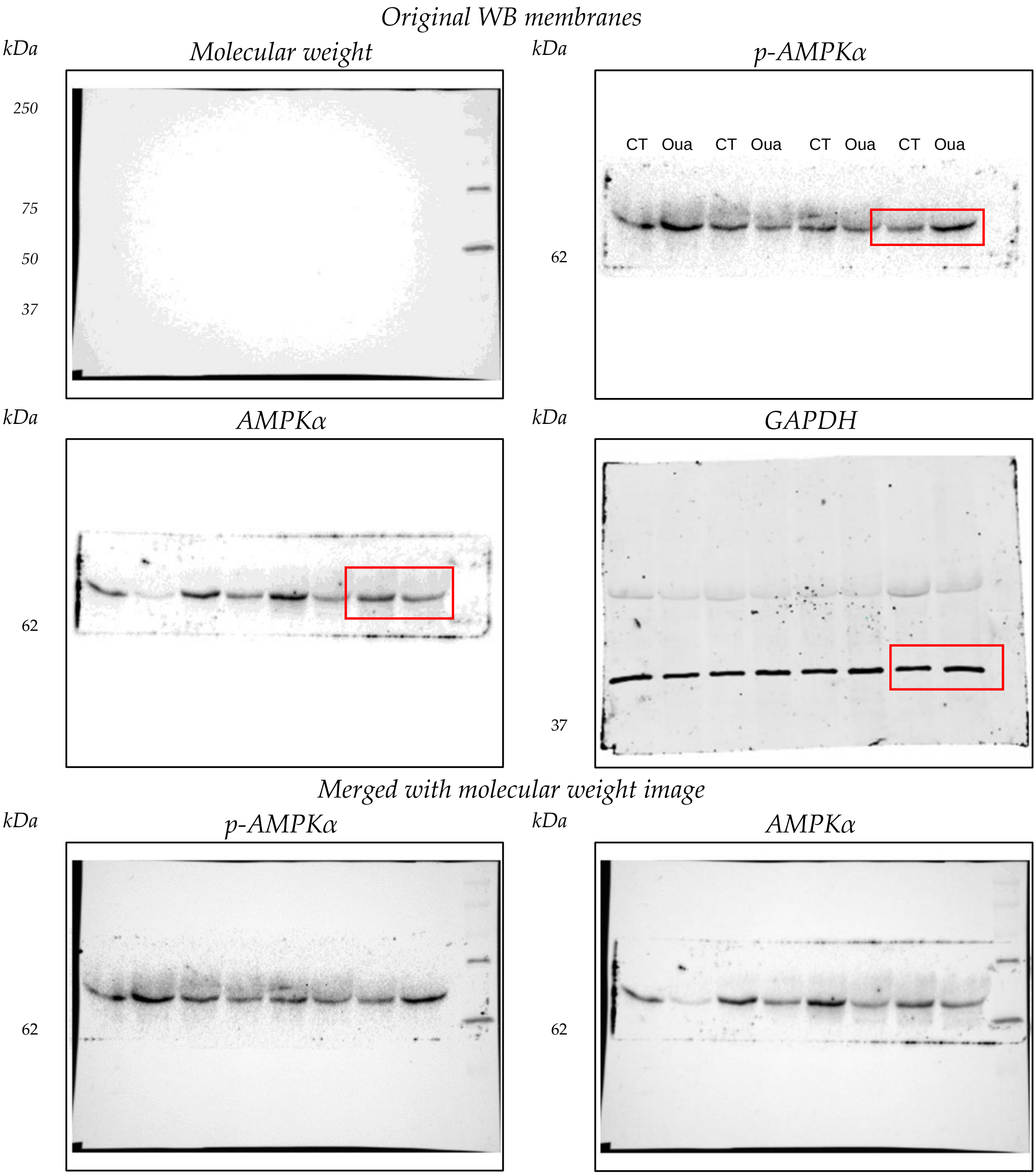
Original WB membranes
kDa
Molecular weight
250
75
50
37
kDa
p-AMPKα
CT Oua CT Oua CT Oua CT Oua
62
kDa
AMPKα
62
kDa
GAPDH
37
Merged with molecular weight image
kDa
p-AMPKα
62
kDa
AMPKα
62

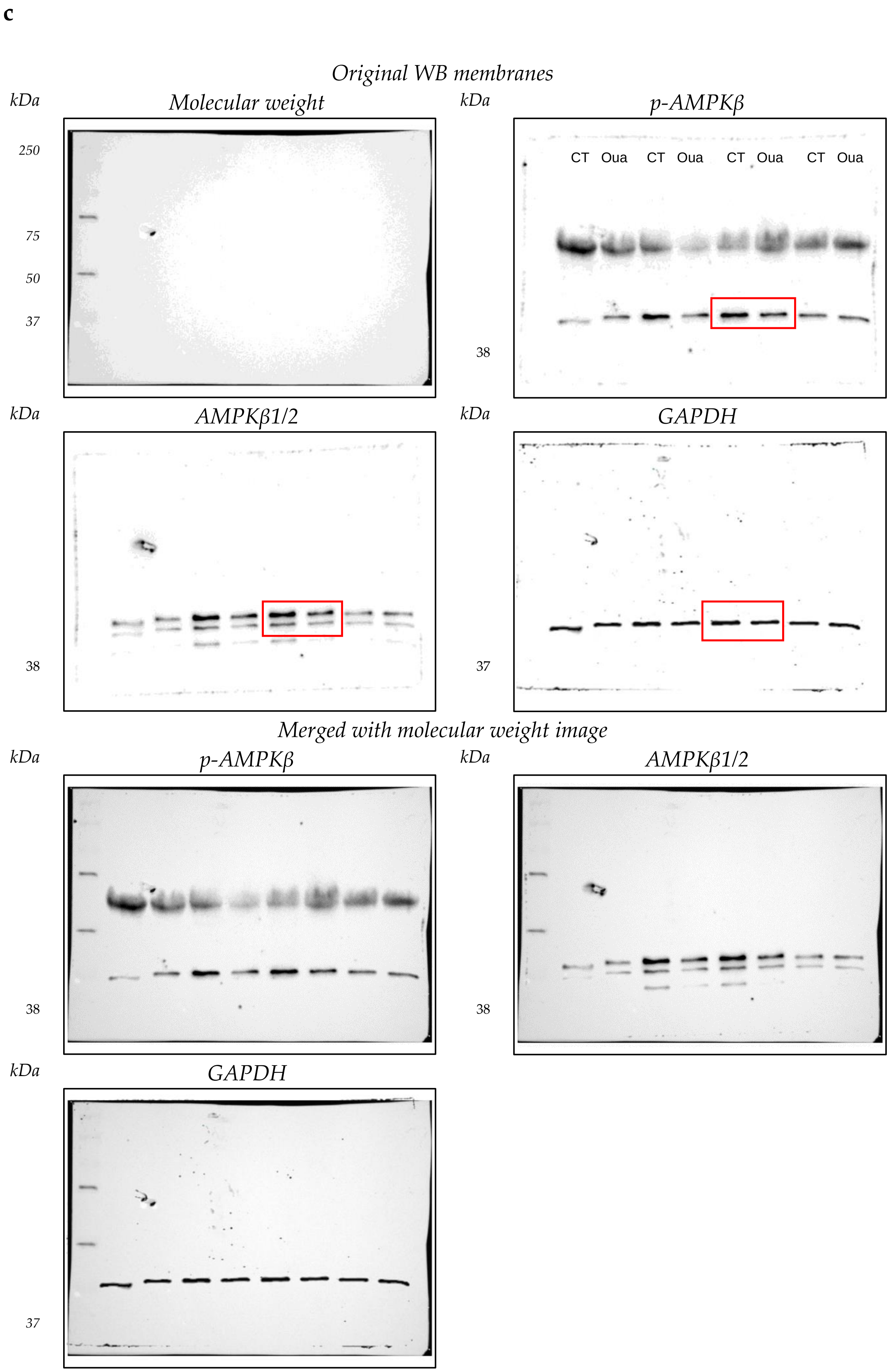
c
Original WB membranes
kDa
Molecular weight
250
75
50
37
kDa
p-AMPKβ
CT Oua CT Oua CT Oua CT Oua
38
kDa
AMPKβ1/2
38
kDa
GAPDH
37
Merged with molecular weight image
kDa
p-AMPKβ
38
kDa
AMPKβ1/2
38
kDa
GAPDH
37

Supplemental figure: Images of original western blots used for preparation of Figure 8.
