# Supplementary material for: Ouabain Effects on Human Anaplastic Thyroid Carcinoma 8505C Cells
Source: Cancers (Basel). 2022 Dec 14;14(24):6168. doi: 10.3390/cancers14246168 (PMC9777381; doi:10.3390/cancers14246168)
Supplement: Supplementary file 1 [file cancers-14-06168-s001.zip › cancers-2101677-supplementary.pdf]

Table S1. Primers used for real-time PCR assay, product size and GenBank accession number.

Gene	Sense	Antisense	Product size (bp)	GenBank accession no.
PAX8	actacaaacgccagaaccc	agggaggttgaatggttgc	149	NM_013992.4
TTF1	atgactggaagacgattggtg	agccttgattagtttccggg	131	NM_007344.4
VIM	cgtgaataccaagacctgctc	ggaaaagtttggaagaggcag	117	NM_003380.5
N-cadherin	cccaagacaaagagacccag	gccactgtgcttactgaattg	140	NM_001792.5
TWIST1	tgtccgcgtcccactagc	tgtccattttctccttctctgga	93	NM_000474.4
MMP9	ttcgacgatgacgagttg	ggattggccttggaagat	285	NM_004994.3
IL-6	caacctgaaccttccaaagatg	acctcaaactccaaaagaccag	108	NM_001371096.1
IL-6R	tcagggttgtggaatcttgc	gagctcaaaccgtagtctgtag	142	NM_181359.3
TGFb1	cctgcccctacatttggag	ccgggttatgctggttgtac	72	NM_000660.7
TGFbRI	acatgattcagccacagatacc	gcatagatgtcagcacgtttg	138	NM_004612.4
TGFbRII	gaaacgatgaaggacaacgtg	cactcagtcaacgtctcacac	107	NM_003242.6
B-actin	actcttccagccttccttcct	cagtgatctccttctgca	176	NM_001101.5
